# Teriflunomide Safety and Efficacy in Advanced Progressive Multiple Sclerosis

**DOI:** 10.1155/2020/5471987

**Published:** 2020-12-17

**Authors:** Vanessa F. Moreira Ferreira, Danielle Caefer, Natalie Erlich-Malona, Brian C. Healy, Tanuja Chitnis, James M. Stankiewicz

**Affiliations:** ^1^Department of Neurology, Brigham and Women's Hospital, Partners MS Center, Harvard Medical School, Boston, MA, USA; ^2^University of Connecticut, Department of Physiology and Neurobiology, Storrs, CT, USA; ^3^Warren Alpert Medical School of Brown University, Department of Neurology, Providence, RI, USA

## Abstract

**Objectives:**

To explore the safety and efficacy profile of teriflunomide in progressive multiple sclerosis.

**Methods:**

We conducted a single-center retrospective observational analysis of a progressive multiple sclerosis population, assessing safety and efficacy in patients treated at least one year with teriflunomide or glatiramer acetate. Sustained progression of expanded disability status scale and sustained worsening of timed 25-foot walk were compared using a Cox proportional hazards model.

**Results:**

Teriflunomide group (*n* = 29) mean characteristics: age = 58 years (SD ± 7.6), disease duration = 16.7 years (SD ± 9.5), expanded disability status score = 5.9 (SD ± 1.3), and follow − up = 32.4 months (SD ± 13.6). Glatiramer acetate group (*n* = 30) mean characteristics: age = 52.4 years (SD ± 11.3), disease duration = 15.1 years (SD ± 10.4), expanded disability status score = 5.7 (SD ± 1.6), and follow − up = 46.9 months (SD ± 43.9). Both treatments were well tolerated without serious side effects. After adjustment for age, sex, and baseline expanded disability status score, sustained expanded disability status score progression did not differ between groups (hazard ratio = 1.17; 95% confidence interval: 0.45, 3.08; *p* = 0.75). Sustained timed 25-foot walk worsening after adjustment also did not differ (hazard ratio = 0.56; 95% confidence interval: 0.2, 1.53; *p* = 0.26).

**Conclusion:**

In an advanced progressive multiple sclerosis population, no substantial differences in tolerability, safety, sustained EDSS progression, or sustained T25FW worsening over time were observed between glatiramer acetate and teriflunomide-treated groups. The small sample precluded definitive determination.

## 1. Introduction

There are many approved agents for treatment of relapsing-remitting multiple sclerosis (MS), but few options exist for patients with progressive forms of MS. Mitoxantrone, though approved for secondary progressive MS, finds limited use due to risks of cardiomyopathy and acute myelogenous leukemia [1]. A phase three trial of ocrelizumab in a primary progressive population achieved a 24% reduction in patients experiencing 12-week confirmed disability progression compared to placebo. The safety profile of ocrelizumab in trial was ostensibly good but requires further definition over time [2, 3]. Siponimod demonstrated efficacy in a phase three trial in secondary progressive multiple sclerosis (SPMS) patients, resulting in a 21% reduction in patients with 12-week confirmed disability progression [4] but also will require further characterization of safety profile over time. Many drugs including interferon beta-1a subcutaneous [5], interferon beta-1a intramuscular [6], glatiramer acetate [7], fingolimod [8], and natalizumab [9] have failed in randomized controlled trials in primary progressive multiple sclerosis (PPMS) or SPMS.

Teriflunomide is an FDA approved medication for relapsing-remitting multiple sclerosis that noncompetitively inhibits dihydroorotate dehydrogenase, a mitochondrial enzyme involved in de novo pyrimidine synthesis, thus halting the cell cycle in S phase and preventing lymphocyte proliferation [10]. In the phase three teriflunomide trial program, a small cohort of PPMS and SPMS patients (*n* = 122, 5.4%) was included [11, 12]. The published trials do not report outcomes in these patient subsets, though the overall trial results including these patient groups as well as relapsing-remitting patients did demonstrate a reduction in relapse rates, improve MRI outcomes, and reduce disability progression compared to placebo. An extension analysis by Nelson et al. [13] of progressive patients included in these trials concluded that treatment may have been beneficial to some patients with progressive MS but this analysis included patients with progressive relapsing MS and did not report results specific to the nonrelapsing progressive population.

Göttle et al. [14] recently reported that teriflunomide promotes oligodendroglial cell differentiation and *in vitro* myelination in rats. In animal models sharing pathogenic similarities with progressive MS patients, teriflunomide also demonstrates some capacity to block ongoing axonal damage allowing for dendritic arborization and neuronal recovery [15]. Retrospective analysis of disease-modifying agent efficacy on short and medium-term disability outcomes in PPMS and SPMS conducted in a large international cohort (MSBase) included few teriflunomide-treated patients but found that MS disease-modifying agents as a whole did not prevent disability progression [16, 17]. Additionally, progressive patients tend to be older and may have a more senescent immune system, potentially increasing susceptibility to infection with immunomodulatory treatments. As such, further characterization of the safety and efficacy of teriflunomide in progressive multiple sclerosis is needed. Here, we report clinical outcomes in progressive multiple sclerosis patients treated at our center with teriflunomide, using glatiramer acetate as a comparator.

## 2. Materials and Methods

This was a single-center retrospective observational analysis of medical records available in the Comprehensive Longitudinal Investigation in MS Brigham and Women's Hospital (CLIMB) database, for progressive patients treated at a tertiary academic center with teriflunomide (2012-2019) or glatiramer acetate (2000-2019). Patients who initiated treatment with teriflunomide or GA after diagnosis of progressive MS (PPMS or SPMS) and treated for at least one year were considered for inclusion. History of relapses in the year prior to drug start was collected to determine disease activity. Teriflunomide and GA are approved medications for the treatment of relapse-remitting MS, and the decision to start these treatments was made by the MS specialist in conjunction with the patient. Patients with noted nonadherence or lacking a clinic visit either three months before or after treatment initiation or whose prior medication (<180 days) could confound the assessment due to enduring effect (cyclophosphamide, mycophenolate, or rituximab) were excluded from the study. Patients were also excluded if they had received ongoing concurrent treatment that could affect progression rate (i.e., disease-modifying agent or recurrent monthly methylprednisolone). Data was collected for each patient at each clinic visit for the duration of treatment with teriflunomide or GA, including dose, side effects, expanded disability status score (EDSS), and timed 25-foot walk (T25FW). Dalfampridine and assistive device use during T25FW was also recorded. Clinic visits were dictated by clinical need but generally occurred every six months.

### 2.1. Definition of Progression

Sustained disability progression was defined as an increase in the EDSS of at least one point from the baseline that was maintained on the next visit at least 90 days later if the baseline score was 5.5 or less or an increase of at least 0.5 points if the baseline score was more than 5.5 [3, 4]. A patient was considered as having sustained T25FW progression event if there was a 20% increase in walk time confirmed at the next visit at least 90 days later. Sustained T25FW worsening was analyzed in two ways: including all visits or excluding visits with a change in dalfampridine use or change in assistive device.

### 2.2. Statistical Analysis

To compare the two groups in terms of the time to disease progression, we evaluated sustained EDSS worsening and sustained T25FW worsening using Cox proportional hazards model. A multivariate analysis controlling for age, sex, and baseline EDSS was performed. We evaluated EDSS change over time comparing patients treated with teriflunomide and patients treated with glatiramer acetate using a linear mixed-effects model. This analysis was also adjusted for age, sex, and baseline EDSS.

## 3. Results

**Figure 1:**
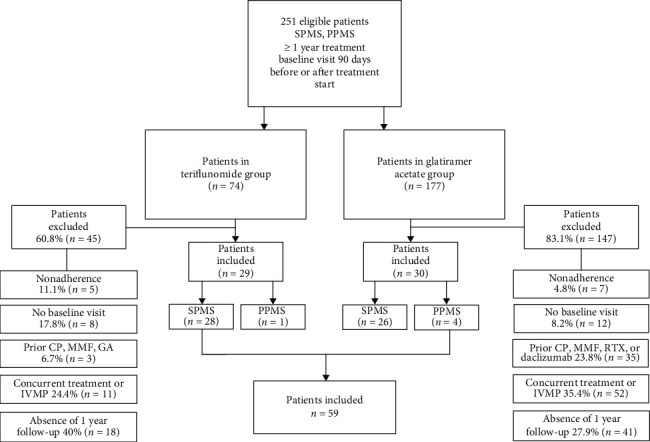
Study flowchart: SPMS: secondary progressive multiple sclerosis; PPMS: primary progressive multiple sclerosis; GA: glatiramer acetate; CP: cyclophosphamide; MMF: mycophenolate; IVMP: intravenous methylprednisolone; RTX: rituximab.

An initial search identified 251 patients with progressive disease in our clinical database: 74 patients treated with teriflunomide and 177 treated with GA. After screening for inclusion/exclusion criteria, fifty-nine patients were included in the study, twenty-nine patients in the teriflunomide group, and thirty patients in the glatiramer acetate group (Figure 1).

Data from two patients were censored after a plausibly unrelated event markedly interfered with their functional status (pulmonary hypertension and mitral valve surgery). Also, data from three patients were censored from the point at which recurrent monthly methylprednisolone infusions were initiated. Population baseline characteristics at the time of either teriflunomide or GA start are reported in Table 1. Relapse history was available in 86.2% (*n* = 25) of patients in the teriflunomide group and 66.7% (*n* = 20) in the GA group, and one patient in each group reported a single relapse within one-year prior treatment start. Baseline age was 57.9 (SD ± 7.6) in the teriflunomide group and 52.4 (SD ± 11.3) in the glatiramer acetate group (*p* = 0.03). The baseline characteristics were not different considering sex (*p* = 0.71), EDSS (*p* = 0.58), T25FW (*p* = 0.11), or disease duration (*p* = 0.54). Prior medication use is listed in Table 2. Patients in the teriflunomide group were treated for a mean of 2.7 years (SD ± 1.1) and GA patients for a mean of 3.9 years (SD ± 3.7).

### 3.1. Tolerability/Safety

No serious safety concerns were identified in either group. Twelve patients in the teriflunomide cohort (41.4%) reported side effects; six patients (20.7%) experienced gastrointestinal discomfort, one of these patients also experienced headache; and hair thinning occurred in three patients (10.3%). Joint pain, fatigue, and anxiety were reported in one patient each. Most patients tolerated the 14 mg dose, though three patients (10.3%) decreased to 7 mg due to side effects. This decreased dose resolved side effects. Five patients in the GA cohort (16.7%) reported side effects related to injection-site pain or reaction. Otherwise, GA was well tolerated.

### 3.2. Efficacy–Clinical Endpoints

**Figure 2:**
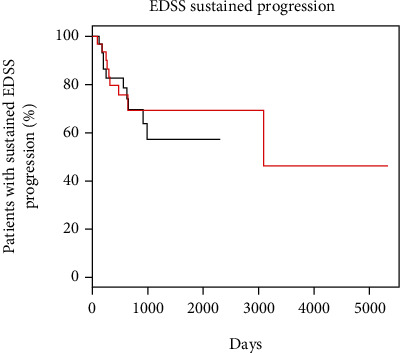
Kaplan-Meier curve for the time to sustained expanded disability status scale (EDSS) progression. Red line represents glatiramer acetate; black line represents teriflunomide. *y*-axis is the probability of remaining free of sustained progression on EDSS, and the *x*-axis is the days since treatment initiation.

Sustained EDSS progression occurred in 34.5% of patients during teriflunomide treatment versus 30% on GA treatment. The risk of sustained disability progression showed a nonsignificant difference between the teriflunomide group and the GA group (HR = 1.17; 95% CI: 0.46, 2.97; *p* = 0.74) (Figure 2). When adjusted for age, sex, and baseline EDSS, the hazard ratio for sustained progression was similar (HR = 1.17; 95% CI: 0.45, 3.08; *p* = 0.75). A linear mixed-effects model showed no difference between patients treated with teriflunomide and GA (difference in EDSS change per year = 0.018; *p* = 0.76) after adjustment for age, sex, and baseline EDSS.

**Figure 3:**
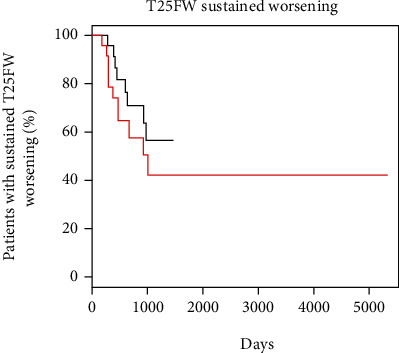
Kaplan-Meier curve of the time to sustained worsening on timed 25-foot walk (T25FW). Red line represents glatiramer acetate; black line represents teriflunomide. *y*-axis is the probability of remaining free of sustained progression on T25FW, and the *x*-axis is the days since treatment initiation.

Baseline T25FW was available for 79.3% (*n* = 23) of patients on teriflunomide and 83.3% (*n* = 25) of patients on GA. There was a limited though nonsignificant difference in the time to sustained worsening of T25FW between teriflunomide and GA (HR = 0.61; 95% CI: 0.25, 1.53; *p* = 0.29) (Figure 3). When adjusted for age, sex, and baseline EDSS, the difference was similar (HR = 0.56; 95% CI: 0.2, 1.53; *p* = 0.26). A sensitivity analysis removing patients experiencing a change in dalfampridine or assistive device use also found no difference between groups (Table 3). Increased assistance with walking occurred in 20.7% of teriflunomide and 20% of GA-treated patients. A decrease in walking assistance occurred in one patient in each group.

## 4. Discussion

The most common side effects reported by the patients in this study were gastrointestinal upset in 20.7% of patients and hair thinning in 10.3% of patients. This is generally consistent with the findings of the TEMSO phase three relapsing-remitting trial, in which diarrhea was reported in 17.9% of patients and hair thinning in 13.1% of the patients receiving the 14 mg dose [11]. These rates were maintained despite the significantly older, more disabled patient population in our study; both the mean age and EDSS were greater than the maximum allowed for inclusion in TEMSO [11]. Safety data captured through visit notes reported that both medications were safe and well tolerated in this older and more disabled cohort.

This study found no significant efficacy differences between the teriflunomide and GA treated cohort in this advanced progressive population. Sustained EDSS progression did not differ between groups when controlling for age, EDSS, and sex. A reduction in hazard (albeit nonsignificant) of T25FW worsening with teriflunomide compared to glatiramer stands in contrast to the EDSS results. Additional study of effects on T25FW with teriflunomide in a larger cohort of patients is warranted.

The pathophysiology of progressive multiple sclerosis likely involves several factors in addition to inflammation, such as mitochondrial dysfunction, iron deposition, and glutamate toxicity disease [18–21]. Additionally, the innate immune system is more active than the adaptive immune system in progressive multiple sclerosis [22–24]. Teriflunomide primarily targets the adaptive immune system in order to reduce the potential for autoreactivity. It, however, does not have a known effect on the innate immune system. It may be that agents acting on the adaptive immune system might have greater efficacy when used in a younger progressive or more inflammatory progressive population. This assertion is supported by the discrepant trial results seen between rituximab and ocrelizumab in PPMS and fingolimod in PPMS and siponimod in SPMS. Ocrelizumab demonstrated a treatment effect vs. placebo in a PPMS population while rituximab did not. The overall trial population for the ocrelizumab PPMS trial compared to the cohort in the rituximab PPMS trial was younger (mean 50 vs. 45 years old, respectively) with shorter disease duration (2.9 vs. 4 years old, respectively) and more patients with gadolinium enhancement at enrollment (27% vs. 25%, respectively) [25]. A similar age was present when comparing the siponimod SPMS and fingolimod PPMS trials (mean age 49 vs. 48 years old) but more patients had baseline gadolinium-enhancing lesions on baseline MRI (21% of patients vs. 14%, respectively) [4, 8]. Our population is older than the ones involved in the clinical trials discussed above.

The literature lacks information about whether teriflunomide might have efficacy in people with progressive multiple sclerosis. This may be because the numbers of patients required to have adequate power to robustly examine this question are difficult to attain in a registry cohort. Our CLIMB cohort is large enough to allow a preliminary exploration of this question after removing noninformative patients. This is a well-characterized patient population observed in a real-life clinical setting, with each treatment group being followed on average over 2.5 years. We believe our results are informative, though acknowledge that we lack sufficient power to offer a definitive determination. We also do not report on MRI results with either medication which might help give additional context to our results. MRIs were not obtained in a homogenous or scheduled way in these patient groups, so they were not included. Additionally, disease activity characterization was limited because relapses within one-year prior treatment start were only partially obtained. It is worth mentioning that a selection bias was potentially introduced by the exclusion of patients that were concomitantly receiving other medications and the exclusion of patients treated with teriflunomide or GA for less than one year. The exclusion criteria were determined in order to prevent possible interference in the teriflunomide or GA efficacy results and to truly represent the efficacy of the medication and the measured outcomes. Finally, patients may have initiated treatment at times when the treatment landscapes differed. The relatively increased availability of new therapies (ocrelizumab, for example) may have led to a lower threshold to switch patients in the teriflunomide group who were perceived to be progressing.

Our choice of glatiramer as a comparator avoids potential confounding by indication bias that might have occurred if we had instead elected to study untreated patients [26]. We chose the GA cohort because it was a homogenous (in contrast to interferon) and large enough group for comparison with adequate follow-up. Also, we opted to compare teriflunomide to a cohort treated with an agent (GA) previously demonstrated to be ineffective in a progressive MS population. A large scale trial (*n* = 943) comparing GA to placebo in a primary progressive population was stopped after an interim analysis determined that GA treatment was ineffective, with patients on GA experiencing EDSS progression as frequently as the placebo-treated group [7]. A post hoc analysis found a discrepant result between male and female patients, with male patients significantly benefitting from GA treatment compared to placebo (HR 0.71; *p* = 0.02) [27]. Though we did control for sex in our analysis, it is also worth noting that a higher proportion of women were included in our cohort compared to the GA PPMS trial (67% female vs. 53% female in PPMS phase three trial), potentially biasing our GA group to have a reduced benefit relative to the phase three trial result if men with progressive disease respond better to GA. Despite a potential observed decrease in efficacy of GA, the risk of sustained disability progression was marginally though nonsignificantly higher for the teriflunomide group rather than the GA group. We believe that the overall negative result seen with GA in PPMS serves as a benchmark that might help inform conclusions about the overall effectiveness of teriflunomide in a progressive population.

Some important caveats of the analysis presented here should be acknowledged, specifically regarding the characteristics of the qualifying P-MS patient cohort. Our population is older (average age in teriflunomide group 58 years old and GA 52 years old), and there was a baseline difference between the two groups. We adjusted for this in our multivariate analysis, but it is possible that our results were skewed in favor of the younger GA-treated group. Also, disease was relatively advanced (mean EDSS 5.8 and 5.7, respectively, in teriflunomide and GA groups), so results may not apply broadly to all patients with progressive multiple sclerosis. Rather, our results are likely to be more informative for a relatively older and more disabled population.

## 5. Conclusion

Both teriflunomide and glatiramer acetate were well tolerated in a group of more disabled progressive patients; our results preliminarily suggest that other medications would be preferred for use in an advanced progressive population given a similarity between patient outcomes demonstrated here and a prior unsuccessful phase three trial of GA in PPMS. However, we acknowledge we have a small sample size that precludes definitive conclusion, and further studies with larger cohorts would be necessary to offer a definitive determination.

## Figures and Tables

**Table 1:** Demographic characteristics at baseline.

Baseline characteristics	Teriflunomide	Glatiramer acetate	*p* value
Patients	29	30	
Mean age, years (SD)	57.9 (7.6)	52.4 (11.3)	0.03
Median age (years)	59.3	55.8	
Female % (*n*)	58.6 (17)	66.7 (20)	0.71
SPMS % (*n*)	96.6 (28)	86.7 (26)	
Mean disease duration, years (SD)	16.7 (9.5)	15.1 (10.4)	0.54
Median disease duration (years)	14.4	12.6	
Mean treatment duration, months (SD)	32.4 (13.6)	46.9 (43.9)	0.09
Median treatment duration, months (SD)	32.9	34.2	
EDSS mean (SD)	5.9 (1.3)	5.7 (1.6)	0.58
EDSS median	6	6	
T25FW mean, seconds (SD)	10.3 (5.7)	15.3 (13.6)	0.11
T25FW median, seconds	8.1	11.6	

SPMS: secondary progressive multiple sclerosis; EDSS: expanded disability status scale; T25FW: timed 25-foot walk; SD: standard deviation; *n*: number of subjects.

**Table 2:** Prior medication.

Prior medication	Teriflunomide *n* = 29 (%)	Glatiramer acetate *n* = 30 (%)
Cyclophosphamide	6 (20.7)	2 (6.7)
Dimethyl fumarate	8 (27.6)	5 (16.7)
Fingolimod	2 (6.9)	0 (0)
Glatiramer acetate	2 (6.9)	2 (6.7)
Interferon beta-1a IM	1 (3.4)	5 (16.7)
Interferon beta-1a SC	1 (3.4)	3 (10.0)
Methotrexate	0 (0)	2 (6.7)
Methylprednisolone IV	2 (6.9)	1 (3.3)
Mycophenolate	1 (3.4)	0 (0)
Natalizumab	2 (6.9)	2 (6.7)
None	2 (6.9)	7 (23.3)
Riluzole	1 (3.4)	0 (0)
Riluzole and interferon beta-1a SC	0 (0)	1 (3.3)
Rituximab	1 (3.4)	0 (0)

IM: intramuscularly; SC: subcutaneously; IV: intravenously; *n*: number of subjects.

**Table 3:** Hazard ratio, 95% confidence intervals, and *p* value for EDSS and T25FW worsening comparing teriflunomide and glatiramer acetate-treated patients.

	Unadjusted		Adjusted^a^	
	HR (95% CI)	*p* value	HR (95% CI)	*p* value
EDSS sustained progression	1.17 (0.46, 2.97)	*p* = 0.742	1.17 (0.45, 3.08)	*p* = 0.745
T25FW sustained worsening	0.61 (0.25, 1.53)	*p* = 0.292	0.56 (0.2, 1.53)	*p* = 0.255
T25FW sustained worsening^b^	0.46 (0.12, 1.77)	*p* = 0.255	0.31 (0.07, 1.38)	*p* = 0.126

EDSS: expanded disability status scale; T25FW: timed 25-foot walk; HR: hazard ratios; CI: confidence intervals. ^a^Adjusted analysis for baseline age, sex, and EDSS. ^b^Sensitivity analysis excluding patients with a change in dalfampridine or assistive device use.

## Data Availability

The data used to support the findings of this study are available from the corresponding author upon reasonable request.
